# Mapping the Allosteric Action of Antagonists A740003 and A438079 Reveals a Role for the Left Flipper in Ligand Sensitivity at P2X7 Receptors[Fn FN3]

**DOI:** 10.1124/mol.117.111021

**Published:** 2018-05

**Authors:** Rebecca C. Allsopp, Sudad Dayl, Anfal Bin Dayel, Ralf Schmid, Richard J. Evans

**Affiliations:** Department of Molecular and Cell Biology (R.C.A., S.D., A.B.D., R.S., R.J.E.) and Leicester Institute of Structural and Chemical Biology (R.S.), University of Leicester, Leicester, United Kingdom; and Department of Chemistry, College of Science, University of Baghdad, Baghdad, Iraq (S.D.)

## Abstract

P2X7 receptor (P2X7R) activation requires ∼100-fold higher concentrations of ATP than other P2X receptor (P2XR) subtypes. Such high levels are found during cellular stress, and P2X7Rs consequently contribute to a range of pathophysiological conditions. We have used chimeric and mutant P2X7Rs, coupled with molecular modeling, to produce a validated model of the binding mode of the subtype-selective antagonist A438079 at an intersubunit allosteric site. Within the allosteric site large effects on antagonist action were found for point mutants of residues F88A, D92A, F95A, and F103A that were conserved or similar between sensitive/insensitive P2XR subtypes, suggesting that these side-chain interactions were not solely responsible for high-affinity antagonist binding. Antagonist sensitivity was increased with mutations that remove the bulk of side chains around the center of the binding pocket, suggesting that the dimensions of the pocket make a significant contribution to selectivity. Chimeric receptors swapping the left flipper (around the orthosteric site) reduced both ATP and antagonist sensitivity. Point mutations within this region highlighted the contribution of a P2X7R-specific aspartic acid residue (D280) that modeling suggests forms a salt bridge with the lower body region of the receptor. The D280A mutant removing this charge increased ATP potency 15-fold providing a new insight into the low ATP sensitivity of the P2X7R. The ortho- and allosteric binding sites form either side of the *β*-strand Y291-E301 adjacent to the left flipper. This structural linking may explain the contribution of the left flipper to both agonist and antagonist action.

## Introduction

ATP is released from cells in different ways, including regulated exocytosis from neurons, following platelet activation, and in response to tissue damage/cell death. It acts as a ligand for P2X-receptor (P2XR) cation channels and a subset of G protein-coupled P2Y receptors (Burnstock, 2012). The contribution of extracellular ATP acting at cell surface P2XRs is now well established in physiologic and pathophysiological contexts ranging from taste sensation to blood clotting (Kaczmarek-Hajek et al., 2012). Within the P2XR family (homo- and heterotrimeric receptors formed from seven P2XR subunits, P2X1–7), the P2X7R is unique, as it has an EC_50_ of ∼0.3–1 mM (at physiologic concentrations of calcium and magnesium), compared with ∼1–30 *μ*M for the other subtypes (North, 2002; Kaczmarek-Hajek et al., 2012). High levels of extracellular ATP are not generally found in healthy tissues, and so the activity of P2X7Rs under normal physiologic conditions is considered to be negligible. Raised extracellular levels (millimolar ATP), however, are found as a response to inflammation, cell damage, and necrosis, resulting in stimulation of P2X7Rs on a variety of cell types, including macrophages, neurons, oligodendrocytes, osteoblasts, fibroblasts, and endothelial and epithelial cells (Bartlett et al., 2014). Activation opens the P2X7R channel pore leading to membrane depolarization as well as the permeation of large cations up to 900 Da, and prolonged stimulation can lead to cell death (North, 2002; Browne et al., 2013). As a result, P2X7Rs are involved in a range of pathophysiological conditions, and selective antagonists have considerable potential in the treatment of a variety of conditions, including inflammation, transplant rejection, pain, and neurologic disorders (Skaper et al., 2010; Sorge et al., 2012; Bartlett et al., 2014). For example, the selective P2X7R antagonist A438079 (3-(5-(2,3-dichlorophenyl)-1H-tetrazol-1-yl)methyl pyridine hydrochloride hydrate) protects against status epilepticus (Engel et al., 2012), ischemic kidney injury (Yan et al., 2015), and colitis (Wan et al., 2016).

The crystallization of the panda (pd) P2X7R in the presence of allosteric inhibitors shows a relaxed interplay of the subunits at the apex of the receptor compared with other P2XR structures currently available (Karasawa and Kawate, 2016). This gives rise to a crevice at the interface of subunits in proximity to but not in direct contact with the ATP binding site. This crevice constitutes the allosteric antagonist binding site for structurally distinct P2X7R-selective antagonists (Karasawa and Kawate, 2016). Prior to the availability of the structures of P2X receptors, a range of mutagenesis studies identified key molecular determinants of receptor properties, including residues involved in ATP action and the location of the channel gate (Chataigneau et al., 2013; Samways et al., 2014). Differences in antagonist action between receptor subtypes and species variants have been used in chimeric and mutagenesis studies to identify residues that are important for drug selectivity at P2XRs that have been used in conjunction with molecular docking (Michel et al., 2009; Wolf et al., 2011; El-Ajouz et al., 2012; Farmer et al., 2015). For the hP2X7R, we used a similar chimera/point mutation and molecular docking approach to map systematically the allosteric binding site for AZ10606120 (*N*-[2-[[2-[(2-hydroxyethyl)amino]ethyl]amino]-5-quinolinyl]-2-tricyclo[3.3.1.13,7]dec-1-ylacetamide dihydrochloride), and these predictions were consistent with the antagonist-bound pdP2X7R crystal structure (Allsopp et al., 2017). Our mutagenesis and simulations also highlighted several features that underlie AZ10606120 selectivity that were not evident from the crystal structure, e.g., the P2X7R unique residues T90 and T94 contribute to the formation/stabilization of the allosteric pocket (Allsopp et al., 2017). To date there is no structural information available for the binding mode of the P2X7R antagonist A438079. The current paper describes our work on A438079 action at the hP2X7R using a combination of chimeras, point mutants, and molecular docking and: 1) characterizes the contribution of regions and residues important for ligand action, and demonstrates an unexpected role of the left flipper as a determinant of reduced ATP sensitivity at the P2X7R; 2) establishes the suitability of molecular docking approaches for P2X7Rs; and 3) compares and classifies the binding modes of three P2X7R-selective antagonists. This work reveals a range of similarities and differences in mode of action as highlighted by analyses of the important contributions of unique, variant, and conserved residues to the allosteric pocket.

## Materials and Methods

### 

#### Pharmacological Characterization of hP2X7Rs.

The generation of the P2X7-2N*β* chimeras and point mutants have been described previously (Allsopp et al., 2017). Additional point mutants were made using the QuikChange mutagenesis kit (Stratagene California, La Jolla, CA). The production of the correct mutations and absence of coding errors was determined by DNA sequencing (Automated ABI Sequencing Service, University of Leicester, UK). cRNA was generated for the mutants and 50 nl (50 ng) was injected into manually defoliculated stage V *Xenopus laevis* oocytes using an Inject+Matic microinjector (J.A. Gabay, Inject+Matic, Geneva, Switzerland). Injected oocytes were stored at 16°C in ND96 buffer [in millimolar concentrations, NaCl 96, KCl 2, CaCl_2_ 1.8, MgCl_2_ 1, sodium pyruvate 5, and HEPES 5 (pH 7.6) supplemented with 50 *μ*g/ml gentamycin and 50 *μ*g/ml tetracycline]. Three to seven days postinjection two-electrode voltage clamp recordings were made from oocytes bathed in divalent-free ND96 buffer (in millimolar concentrations, NaCl 96, KCl 2, sodium pyruvate 5, HEPES 5, and 0.1 flufenamic acid, pH 7.6). Oocytes were voltage clamped at a holding potential of −60 mV with a GeneClamp500B amplifier (Molecular Devices).

Electrophysiological traces were digitized with a Digidata 1322A and collected using pCLAMP 8.2 software (Molecular Devices, Menlo Park, CA). An EC_90_ concentration of ATP was used to test antagonist sensitivity for the P2X7-2N*β* and mutant receptors [ATP sensitivity of the chimeras and mutants are reported in Allsopp et al. (2017)] to standardize for any changes in ATP sensitivity. ATP was applied via a U-tube perfusion system for 3 seconds at 3- to 5-minute intervals to allow reproducible responses to be recorded. Antagonists (A438079 or A740003; Tocris/Bio-Techne Corporation, Minneapolis, MN) were bath-perfused as well as coapplied with ATP through the U tube.

#### Molecular Modeling.

Homology models of the hP2X7R trimer in the closed form were built using as a basis the X-ray structures of the pdP2X7R closed forms (PDB ID 5U1L, 5U1U, 5U1V, 5U1W, 5U1X, and 5U1Y) and a multiple template approach in MODELER 9.15 (Webb and Sali, 2016). Redocking experiments, i.e., removing the antagonist from the X-ray structure and docking it back, were performed in RosettaLigand (Davis and Baker, 2009) for all available antagonist-bound pdP2X7R structures (5U1U, 5U1V, 5U1W, 5U1X, and 5U1Y). Rosetta was also used for ensemble docking of the antagonist A438079, for which no X-ray structure is available, into hP2X7R. In the docking protocol, the allosteric site was defined by a 12-Å sphere centered at the C*β* atom of D92, the orthosteric site by a 12-Å sphere centered at the C*β* atom of K64. Ten representative starting structures for ensemble ligand docking were derived from 50-nanosecond molecular dynamics simulations of hP2X7R models. Molecular dynamics simulations of hP2X7R and hP2X7R D92A embedded in a DMPC bilayer were performed in Amber 16 (Case et al., 2017) using ff14SB and lipid14 force fields and a setup described previously (Allsopp et al., 2017). Analysis of molecular dynamics trajectories and RosettaLigand docking results followed the protocol established for AZ10606120 (Allsopp et al., 2017).

#### Data Analysis.

Inhibition by the antagonists was expressed as the percentage of the peak current amplitude of an EC_90_ concentration of ATP recorded before the application of antagonist (ATP gave reproducible responses to ATP in the absence of antagonist). Inhibition curves were fitted with the Hill equation (variable slope) using GraphPad Prism 6 (GraphPad Software, San Diego, CA). IC_50_ is the concentration of antagonist required to inhibit by 50% the response to an EC_90_ concentration of ATP. pIC_50_ is −log_10_ of the IC_50_ value. Individual concentration-response curves were generated for individual experiments, and statistical analysis was carried out on the data generated. When shown in figures, the inhibition curves are fitted to the mean normalized data. Any significant differences from the P2X7-2N*β* control were calculated by one-way analysis of variance, followed by Dunnett’s test (using GraphPad Prism 6). Data are shown as mean ± S.E.M. In all cases *n* ≥ 3 for all data points.

## Results

### 

#### Similarities and Differences in the Mode of Action of P2X7R Antagonists Revealed with Chimeric Receptors and Deletion of a P2X7R Unique Insertion.

**Fig. 1.**
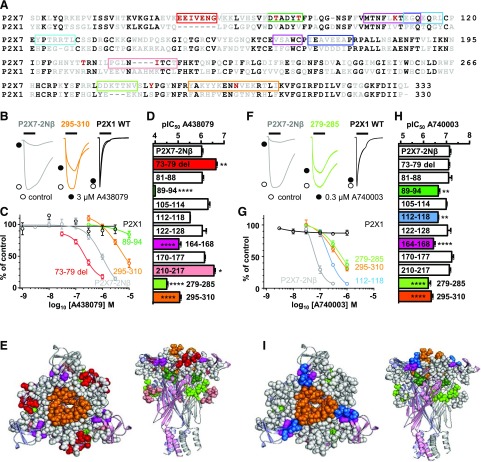
Chimeric hP2X7-1 receptors identify regions important for action of the P2X7R-selective antagonists A438079 and A740003. (A) Sequence alignment of the extracellular loop of the human P2X1 and P2X7Rs. Residues in black are conserved between human P2XR paralogs, gray residues are variant between human P2XR paralogs, and red residues are unique to the P2X7R. Colored boxes show the regions of P2X7 that were replaced with the corresponding residues from the P2X1R to generate the chimeras. (B) Effects of the antagonist A438079 (3 *μ*M, traces indicated by black circles) on currents evoked by an EC_90_ concentration of ATP (3s application indicated by black bar) at the P2X7-2N*β*, 279–285 chimera and P2X1R. Control responses are indicated by open circles. (C) Concentration-dependent inhibition by A438079 of responses to an EC_90_ concentration of ATP for P2X7-2N*β* (gray), 73–79 del (red), chimeras 295–310 (orange), 279–285 (light green), and P2X1 (black). (D) Histogram showing the pIC_50_ of A438079 at P2X7-2N*β* and chimeric receptors. (E) Location of chimeras that reduced A438079 action mapped on a pdP2X7R-based homology model. Chimeras with no change are shown as gray spheres. (F) Effects of the antagonist A740003 (0.3 *μ*M, traces indicated by black circles) on currents evoked by an EC_90_ concentration of ATP (3s application indicated by black bar) at the P2X7-2N*β*, 295–310 chimera and P2X1R. Control responses are indicated by open circles. (G) Concentration-dependent inhibition by A740003 of responses to an EC_90_ concentration of ATP for P2X7-2N*β* (gray), chimeras 112–118 (light blue), 295–310 (orange) and 89–94 (green), and P2X1 (black). (H) Histogram showing the pIC_50_ of A740003 at P2X7-2N*β* and chimeric receptors. (I) Location of chimeras with an effect on A740003 action mapped on a pdP2X7R-based homology model. Chimeras with no change are shown as gray spheres. *n* ≥ 3, exact values given for each receptor tested are shown in Table 1. *P < 0.05; **P < 0.01; ****P < 0.0001.

At the P2X7-2N*β* receptor, A438079 inhibited ATP (EC_90_ concentration)-evoked currents in a concentration-dependent manner (pIC_50_ of 6.03 ± 0.05) consistent with previous studies (Nelson et al., 2006) and was ineffective at the hP2X1R (Fig. 1B–E; Table 1). The P2X7-2N*β* receptor has residues 16–26 of the intracellular amino terminus replaced by those from the P2X2R and this allows reproducible stable ATP-evoked responses to be recorded with no effect on the pharmacological properties of the P2X7R. We have reported the effects on ATP action of a range of point mutants and chimeric receptors (Allsopp et al., 2017) on the basis of the P2X7-2N*β* receptor swapping regions around the orthosteric and allosteric binding sites of the hP2X7R with the corresponding regions of the hP2X1R. In our studies with chimeras (and point mutants) an EC_90_ concentration of ATP was used to standardize the testing of the effects of antagonists (El-Ajouz et al., 2012; Farmer et al., 2015; Allsopp et al., 2017). To determine where the antagonist A438079 acts, we determined the sensitivity of the antagonist at a range of chimeric P2X7/1 receptors (Fig. 1B–E; Table 1). Less than 3-fold changes in antagonist sensitivity were not considered important (Allsopp et al., 2017).

**TABLE 1:** Antagonist sensitivity at chimeric P2X7Rs *RESIZE*Mean pIC_50_ values ± S.E.M. and fold change in sensitivity relative to P2X7-2N*β* are shown for the antagonists A740003 and A438079 at chimeric P2X7Rs. *n* numbers are shown for each receptor tested.

	A740003		A438079	
	*pIC*_50_ *± S.E.M.*	*n*	*pIC50 ± S.E.M.*	*n*
P2X7-2N*β*	7.1 ± 0.05	6	6.0 ± 0.05	4
73–79	7.1 ± 0.04	3	6.7 ± 0.05**	6
81–88	7.1 ± 0.13	3	6.0 ± 0.06	3
89–94	6.6 ± 0.06**	3	<4****	3
105–114	7.0 ± 0.04	3	6.3 ± 0.08	4
112–118	6.6 ± 0.02**	5	5.5 ± 0.01	3
122–128	7.0 ± 0.14	3	6.1 ± 0.18	4
164–168	6.4 ± 0.07****	4	5.0 ± 0.09****	3
170–177	7.2 ± 0.08	3	6.2 ± 0.03	3
210–217	7.1 ± 0.05	3	6.6 ± 0.06*	4
279–285	6.2 ± 0.10****	3	4.5 ± 0.05****	4
280–284 of P2X4	6.1 ± 0.05****	3	4.5 ± 0.05****	3
295–310	6.3 ± 0.07****	3	5.0 ± 0.06****	3

* *P* < 0.05;

** *P* < 0.01;

**** *P* < 0.0001.

The P2X7R has a unique insertion (residues EEIVENG) and a four-amino-acid deletion in the dorsal fin (encompassed by chimera 210–217). Removal of the insertion (73–79 deletion) increased A438079 sensitivity ∼4-fold, and there was an ∼3-fold increase in sensitivity at the 210–217 chimera. This contrasts with the effects we have reported previously for the allosteric antagonist AZ10606120 at these chimeras (40-fold decrease and no change, respectively) (Allsopp et al., 2017). Our molecular modeling (see later) favored allosteric poses for A438079 but also indicated the potential of orthosteric poses. To test whether the changes in sensitivity at chimeras seen for AZ10606120 were a hallmark of an allosteric antagonist, and could therefore help discriminate binding modes, we determined the effects of the chimeras on another allosteric antagonist, A740003 (*N*-[1-[[(cyanoamino)(5-quinolinylamino)methylene]amino]-2,2-dimethylpropyl]-3,4-dimethoxybenzeneacetamide), of which a crystal structure shows binding at the allosteric pocket (Honore et al., 2006; Karasawa and Kawate, 2016). Sensitivity to A740003 (pIC_50_ at P2X7-2N*β* 7.13 ± 0.05 consistent with previous studies) was unaffected by either the 73–79 deletion or 210–217 chimeras. This is an “intermediate” profile with the lack of effect of the 73–79 deletion mutation similar to A438079, whereas the lack of effect of the 210–217 chimera was similar to that for AZ10606120. We were therefore interested to measure sensitivity to both A438079 and A740003 by the range of chimeras we had in order to make comparisons. The chimeras 81–88, 105–114, 122–128, and 170–177 had no effect on the potency of either A438079 or A740003. There was a modest ∼3-fold decrease in sensitivity for both antagonists at the 112–118 chimera (Fig. 1). The 164–168 chimera (adjacent to the top of the allosteric pocket) and chimera 295–310 had similar effects and reduced antagonist sensitivity ∼5- and 10-fold for A740003 and A438079, respectively (Fig. 1). The chimera 89–94, which corresponds to mutation of the two unique threonine residues (T90 and T94) to valines found in all other P2XRs, had a modest ∼3-fold reduction in sensitivity to A740003. In contrast, A438079 at 10 *μ*M only inhibited ATP responses at the 89–94 chimera by 15.7% ± 5.2%, indicating a >1000-fold decrease in affinity (Fig. 1). Our results indicate that chimeras/variations around the allosteric pocket have an effect on the action of A430007 and A438079 and show that there are similarities and differences between the modes of binding/action of these antagonists.

#### Contribution of the Left Flipper to Ligand Action.

**Fig. 2.**
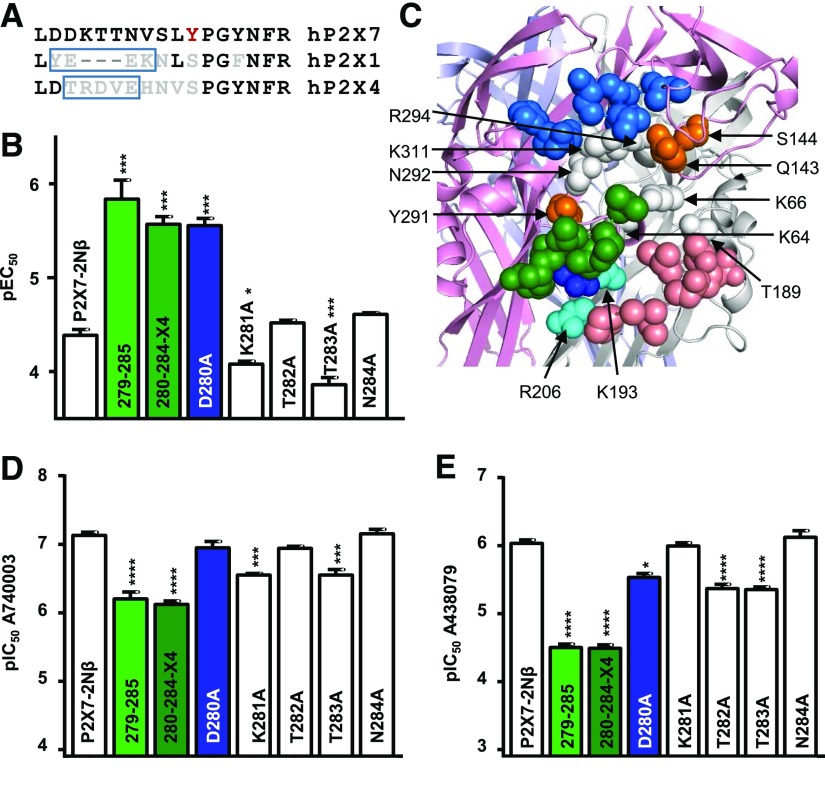
Contribution of the left flipper and variant residues around the orthosteric binding site to ligand action. (A) Sequence alignment of hP2X7R, 1R, and 4R showing the three-residue deletion associated with the P2X7 279–285 chimera (box in hP2X1R sequence) and the region changed in the 280–284-X4 chimera (box in hP2X4R sequence). Residue coloring as in Fig. 1A. (B) Effects of chimeras and point mutations of the left flipper on ATP sensitivity. Data for P21X7-2N*β* and 279–285 taken from (Allsopp et al., 2017). (C) Homology model of the hP2X7R showing the left flipper (green) and region around the orthosteric binding site. Residues coordinating the binding of ATP that are conserved throughout the P2XR family are shown in white. The chimera swapping residues 170–177 (marine blue) just above the agonist site had no effect on sensitivity to A740003 or A438079. Orange residues are variant between P2X7R and P2X1R but when mutated had no effect on antagonist sensitivity. The green region corresponds to left flipper (279–285). The chimera 210–217 (salmon) increased A43 sensitivity. The dark blue residue is D290 and the cyan residues correspond to R206 and K193. (D and E) Effects of chimeras at the left flipper and alanine point mutations of the flipper on sensitivity to A740003 (D) and A438079 (E). Data are shown as mean ± S.E.M., *n* = 3, *P < 0.05; ***P < 0.001; ****P < 0.0001.

In our previous work (Allsopp et al., 2017), the chimera changing a variant region in the left flipper (279–285) to the corresponding section of the P2X1R (DDKTTNVS replaced by YEEK) increased ATP sensitivity, implying an effect on agonist binding/gating but had no effect on AZ10606120 action. This is consistent with its location around the orthosteric binding site and a role in conformational changes in the process of agonist binding (Allsopp et al., 2017). We were therefore surprised to find that the 279–285 chimera reduced antagonist sensitivity by ∼9- and 34-fold for A740003 and A438079, respectively (Fig. 1). This chimera not only changes the residues contributing to the left flipper, including D280 and N284 that are unique to the P2X7R, but also removes three amino acids (Fig. 2). To determine whether the shortening of the flipper loop by removal of the three residues accounts for the decrease in sensitivity, we generated a chimera in which the P2X7R left flipper region 280–284 was replaced by the equivalent region from the P2X4 receptor (residues TRDVE) (Fig. 2). Interestingly, the ATP sensitivity of this chimera was increased ∼15-fold, and it showed as well a decrease in antagonist sensitivity (∼10- and 34-fold decrease for A740003 and A438079, respectively) similar to that of the equivalent P2X1R region swap. This suggests that the variant/unique residues in the 280–284 section contribute to ATP and antagonist sensitivity.

To determine the contribution of individual residues in the left flipper to ligand action, we generated individual alanine mutants for residues 280–284. The D280A mutant showed the largest individual increase in ATP potency (15-fold), mutations of T282 and N284 had no effect on ATP sensitivity, and there was a 3.4- and 2-fold decrease for T283A and K281A mutants (Fig. 2). The homology model of the hP2X7R shows that D280A (a unique residue to P2X7Rs) is close to R206 and K193 and could potentially form a salt bridge with either of these positively charged residues, stabilizing the conformation of the left flipper and thus affecting gating. To test this, we generated alanine mutants at these positively charged residues. ATP sensitivity was not changed at the R206A mutant (pEC_50_ 4.33 ± 0.06), suggesting that it does not form a salt bridge with D280. At the K193A mutant ATP-evoked responses were very small in amplitude (<100 nA), which made estimation of ATP sensitivity difficult and was consistent with a contribution of this conserved residue to the ATP binding site. The potential of a salt bridge between D280 and K193 is supported by molecular dynamics simulations of the hP2X7R in the closed state. The D280/K193 salt bridge was present in 60% ± 5% of all frames, whereas for D280/R206 this was only the case for 28% ± 5%.

The point mutations D280A, T282A, and N284A had no effect on A740003 sensitivity, and there was a modest ∼4-fold decrease for substitutions K281A and T283A. For A438079 the N284A mutation also had no effect on antagonist sensitivity, suggesting that this unique P2X7R residue, which is glycosylated (Lenertz et al., 2010), does not contribute to the action of the antagonists. The K281A mutation also had no effect on A438079 action. In contrast, A438079 affinity was reduced ∼4.5-fold for the mutants T282A and T283A, and at the D280A mutant there was an ∼3-fold decrease in sensitivity. These results indicate that no individual residue in the left flipper makes a major contribution to the antagonist action of A438079 or A740003, but the combination of different residues can affect the properties of the left flipper and antagonist action.

#### A438079 Does Not Act/Bind at the Orthosteric Site.

There are no P2X7R structures with A438079 bound, so its site of action remained to be established. Although our molecular docking pointed toward an allosteric binding mode (see section below), it did not rule out an orthosteric mode of action for A438079. The studies with the chimeras suggested that the site would probably be allosteric (five chimeras affected) rather than orthosteric, as the only “orthosteric” chimera, 279–285, that reduced A438079 action also produced a significant reduction for the known allosteric antagonist A740003. In addition, the chimera 89–94, a proposed modifier of the allosteric pocket (Allsopp et al., 2017), had the greatest effect on sensitivity to A438079. However, for completeness we tested the contribution of three residues around the orthosteric pocket that are unique to the P2X7R (H62, Q143, and the aromatic Y288). When individually mutated to the equivalent residue in the hP2X1R (H62S, Q143K, and Y288S), they either increased A438079 sensitivity modestly (∼3.7-fold for Y288S, *P* < 0.05) or had no effect (H62S and Q143K). Given the limited or lack of effect of mutations of these P2X7R unique residues (and considerably greater effects of allosteric site mutants—see later) and the high degree of conservation of residues forming the ATP binding pocket (Fig. 2), it seems improbable that the A438079 binds at the orthosteric site.

#### Evaluation of Ligand Docking to P2X7Rs.

**Fig. 3.**
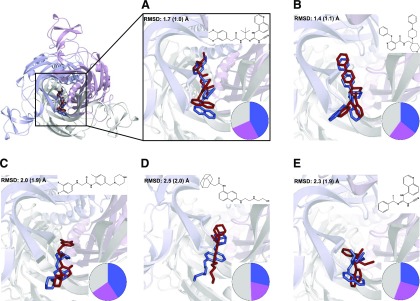
Redocking of allosteric antagonists into pdP2X7R using RosettaLigand. (A) A740003, (B) JNJ47965567, (C) GW791343, (D) AZ10606120, and (E) A804598. (A–E) Reference antagonist poses from X-ray structures 5U1U (A), 5U1X (B), 5U1Y (C), 5U1W(D), and 5U1V(E) are shown as blue sticks. For each antagonist one representative pose from each of the two main clusters in the redocking process was selected. From these two the pose closest to the X-ray structure is shown as red sticks. Pie charts indicate the size of two main clusters from the respective docking run (marine and magenta) with the remaining poses colored in gray. Root-mean-square deviations (rmsd) between the representative docked pose and antagonist in X-ray structures are given in the top left of each panel. The values in brackets refer to the pose with the smallest rmsd within the selected cluster. The pdP2X7R structure is shown in cartoon representation with the three subunits shown in gray, light blue, and pink.

**Fig. 4.**
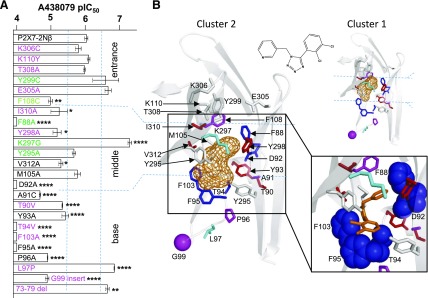
Effects of point mutants in the allosteric binding pocket on sensitivity to the antagonist A438079. (A) Effects of point mutations on A438079 sensitivity (pIC_50_ value). Blue dotted lines correspond to a 3-fold change in sensitivity. Pink residues are variant, green have similar properties, and those in black are conserved between P2X and P2X7Rs. *n* ≥ 3, exact values for each receptor tested are given in Table 2. *P < 0.05; **P < 0.01; ****P < 0.0001. (B) Representative A438079 poses derived from clusters 1 and 2. A438079 is shown in orange mesh in the main panels and orange sticks in the enlarged representation of cluster 2, point mutants that had no or <3-fold shift in A438079 sensitivity are shown in gray, 3–10-fold shift in red, 10–30-fold shift in magenta, >30-fold shift in blue, and an increase in sensitivity in cyan.

The publication of pdP2X7R X-ray structures with five bound allosteric antagonists (A740003, A804598, AZ10605120, GW791343, and JNJ47965667) enabled us to evaluate our RosettaLigand ligand-docking approach by unbiased redocking of these compounds (Fig. 3). For all five test cases the poses most like the experimentally determined structures were found in the two top-ranked clusters of the redocking experiment. The ligand root-mean-square deviations for representative structures from these clusters ranged from 1.4 to 2.5 Å; the solutions closest to the X-ray structures were in the range of 1.0–2.0 Å (Fig. 3). These results validated our ligand-docking approach and demonstrated that we would probably identify poses conformationally close to experimental structures in the top-ranked clusters. For these five antagonists we also compared the mean of the RosettaLigand interface_delta_X docking scores (a measure typically used for ranking RosettaLigand docking poses) of the biggest clusters for docking to the allosteric and orthosteric binding sites. In all five cases the scores for the allosteric binding sites were better (more negative), with differences in scores ranging from 1.9 to 3.6, suggesting that, with available data as a basis, binding modes might be distinguished by comparing scores for allosteric and orthosteric binding sites. For the antagonist A438079, no experimental structure is available. Docking A438079 into the hP2X7 allosteric and orthosteric binding sites results in mean interface_delta_X for the biggest allosteric and orthosteric clusters of −11.3 and −10.5, respectively. The lower score for the allosteric site is pointing toward an allosteric binding mode, though considering the bigger differences seen in the confirmed allosteric compounds above, this may not be conclusive from docking alone. However, as experiment and modeling indicate an allosteric binding mode of A438079, the two main clusters of allosteric binding modes (Fig. 4) were analyzed in the context of the effects of mutating individual residues on antagonism (see below).

#### Identification of Residues Lining the Allosteric Pocket That Are Important for the Antagonist Action of A438079.

**Fig. 5.**
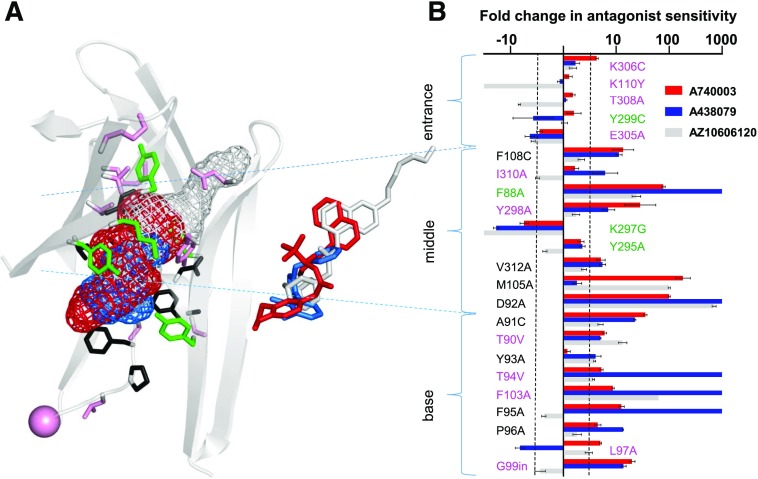
Comparison of effects of point mutations on the allosteric binding pocket in the sensitivity to the P2X7R antagonists A740003, A438079, and AZ10606120. (A) hP2X7R homology model of the allosteric binding pocket with the three antagonists A740003 (red), A438079 (blue), and AZ10606120 (gray) shown in mesh representation. Pink residues are variant, green have similar properties, and those in black are conserved between P2X and P2X7Rs [same color coding as used in (B)]. Stick representation the overlap of the three antagonists bound to the receptor. (B) Graph showing the fold shift in antagonist sensitivity (log scale) for the three P2X7R antagonists (data are shown as mean ± S.E.M. of fold shift relative to P2X7-2N*β*). Data for AZ10606120 taken from reference (Allsopp et al., 2017). Dotted black line indicates a 3-fold difference in antagonist sensitivity. *n* ≥ 3, exact values for A740003 and A438079 are shown in Table 2.

The allosteric binding pocket can be divided into three sections: entrance, middle, and base (Fig. 4). The chimeras investigated the effects of replacing regions of the hP2X7R with the corresponding part of hP2X1 in and around the allosteric pocket and highlighted that variations in this region contribute to A438079 action (Fig. 1). However, there are also several conserved/similar residues in the regions swapped in the chimeras (Fig. 1A). Therefore, we used a point mutagenesis approach to investigate the role of individual residues that contribute to the formation of the allosteric pocket. Where the residue was different between P2X7 and P2X1 the corresponding P2X1R residue was introduced, where the residue was similar/conserved between P2X1 and 7Rs it was mutated to alanine or cysteine. We recently reported the effects of these mutants on ATP and AZ10606120 sensitivity (Allsopp et al., 2017). These mutants have now been tested to determine the contribution of defined residues to the antagonist actions of A438079 (Figs. 4 and 5; Table 2). For comparison, effects on A740003 were also tested (Supplementary Fig. 1).

**TABLE 2:** Antagonist sensitivity at point mutants lining the allosteric antagonist pocket of P2X7Rs The left-hand column shows whether the residues are conserved, similar, or variant between P2X1 and P2X7Rs. Mean pIC_50_ values ± S.E.M. and fold change in sensitivity are shown for the antagonists A740003 and A438079 at P2X7R point mutants. *n* numbers shown for each receptor tested.

		A740003 pIC_50_	*n* =	A438079 pIC_50_	*n* =
	P2X7-2N*β*	7.1 ± 0.05	6	6.0 ± 0.05	4
Variant	H85P	6.4 ± 0.02****	3	6.0 ± 0.02	3
Variant	S86A	7.1 ± 0.03	3	6.0 ± 0.06	3
Variant	T90V	6.4 ± 0.04****	3	5.3 ± 0.01****	3
Variant	T94V	6.4 ± 0.03****	3	<4****	3
Variant	L97P	6.4 ± 0.02****	3	6.9 ± 0.01****	3
Variant	Insert G99	5.8 ± 0.05****	3	4.9 ± 0.05****	3
Variant	F103A	6.2 ± 0.02***	3	<4****	3
Variant	K110Y	7.0 ± 0.06	3	6.1 ± 0.04	3
Variant	S165F	6.7 ± 0.03	3	5.1 ± 0.05**	3
Variant	A166G	6.5 ± 0.02*	3	5.4 ± 0.15	3
Variant	Y298A	5.7 ± 0.30****	3	5.2 ± 0.12*	3
Variant	V304C	7.0 ± 0.02	3	7.2 ± 0.16***	3
Variant	E305A	7.6 ± 0.06	3	6.7 ± 0.09	4
Variant	K306C	6.5 ± 0.03*	3	5.8 ± 0.08	4
Variant	T308A	7.0 ± 0.04	3	6.0 ± 0.02	3
Variant	I310A	6.9 ± 0.07	3	5.2 ± 0.24*	3
Similar	F88W	6.8 ± 0.08	3	5.5 ± 0.03****	3
Similar	F88A	5.3 ± 0.03****	3	<4****	3
Similar	Y295A	6.8 ± 0.06	3	5.7 ± 0.05	3
Similar	K297G	7.9 ± 0.08**	4	7.3 ± 0.05****	3
Similar	Y299C	7.0 ± 0.13	3	6.6 ± 0.38	4
Conserved	L83A	6.5 ± 0.02****	3	6.1 ± 0.05	3
Conserved	A91C	5.6 ± 0.03****	3	4.7 ± 0.02****	3
Conserved	D92A	5.1 ± 0.02****	3	<4****	3
Conserved	Y93A	7.0 ± 0.05	3	5.4 ± 0.10****	3
Conserved	F95A	6.0 ± 0.05****	3	<4****	4
Conserved	P96A	6.5 ± 0.06****	3	4.9 ± 0.01****	3
Conserved	M105A	4.9 ± 0.14****	3	5.8 ± 0.09	3
Conserved	F108C	6.0 ± 0.20****	3	5.0 ± 0.06**	3
Conserved	Q116A	6.5 ± 0.04*	3	5.9 ± 0.05	4
Conserved	V312A	6.4 ± 0.08**	3	5.3 ± 0.06*	3

* *P* < 0.05;

** *P* < 0.01;

*** *P* < 0.001;

**** *P* < 0.0001.

At the entrance to the allosteric pocket are two residues (K110 and K306) unique to the human, rat, and panda P2X7Rs. The K306C and K110Y mutants had no effect on A438079 sensitivity. The remainder of the residues were either variant (T308 and E305) or similar to those found in the hP2X1R (Y299). There was no significant decrease in antagonist sensitivity at T308A, Y299C, or E305A. These results are consistent with the lack of interaction predicted by the ligand docking.

The middle region comprises nine residues that line the pocket. Of these, only two show marked variations in their side chain between hP2X7 and 1 (Y298 and I310). For Y298, tyrosine is at this position for P2X2–5 and 7Rs, and it is histidine for the hP2X1R. The alanine mutation (Y298A) reduced A438079 sensitivity ∼7-fold. At the I310A mutant there was a decrease of ∼6-fold for A438079. Interestingly, there were greater changes in antagonist sensitivity for residues that were conserved or had similar properties. Three residues have similar properties at hP2X1 and hP2X7Rs (F88, Y295, and K297). Removing the aromatic residue at position 88 (F88A) reduced the sensitivity to A438079 by >1000-fold. However, the more conservative mutation, F88W, swapping between the P2X7 and P2X1 side chain had no effect on the sensitivity to the antagonist (data not shown). This indicates that the variation of this residue between P2X1R and P2X7R probably is not a contributor to the differences in antagonist sensitivity, although it does raise the possibility that an aromatic residue at this position is important for high affinity binding of A438079. Mutations to remove the bulk and charge of the side chain at K297 (K297G) increased sensitivity to A438079 ∼19-fold, suggesting that this improves the space/access within the pocket. The Y295A mutant to remove the conserved aromatic group at position 295 (tyrosine at hP2X7R and phenylalanine at all other P2XRs, except the nonfunctional P2X6R) had no effect on antagonist sensitivity.

There are three residues in the middle region that are conserved between the hP2X1R and 7R (M105, F108, and V312) and one residue that is conserved among all mammalian P2XRs (D92). Removal of the aromatic group at position 108 (F108C) reduced antagonist action of A438079 ∼11-fold. The valine to alanine mutation at position 312 (V312A) had a modest ∼5-fold reduction in antagonist sensitivity. Mutation of the methionine at position 105 to alanine had no effect on A438079 sensitivity. Removal of the negatively charged aspartic acid that is conserved in mammalian P2X7Rs at position 92 (D92A no change in ATP sensitivity) reduced antagonist sensitivity by >1000-fold for A438079.

Residues F95 (conserved hP2X1 and 7) and F103 (valine hP2X1) line the base of the allosteric pocket. Alanine mutants of these aromatic residues decreased A438079 sensitivity >1000-fold. The majority of residues that form the base region do not directly line the allosteric binding region but probably influences the folding/dimensions of the pocket, e.g., the unique threonine residues at positions 90 and 94 (Allsopp et al., 2017). The base of the allosteric pocket is formed from an *α*-helix incorporating T90, A91 (D92 facing up and lining the middle section), Y93, and T94, followed by a *β*-strand (with F95 facing the pocket) that loops further down and “turns” at G99 and then forms a *β*-strand that comprises the other side of the base (with F103 facing the antagonist pocket). Mutations of residues that form the *α*-helix at the base of the allosteric pocket reduced antagonist sensitivity 4-fold for A438079. At T90V there was a modest ∼5-fold decrease in sensitivity for A438079 and >20-fold decrease was recorded at A91C. At the T94V mutant there was a >1000-fold decrease for A438079. These results show that residues within the *α*-helix that are not directly accessible are important for shaping the allosteric pocket. Mutations of the loop region connecting the *α*-helix and the *β*-strand residues that form the bottom of the allosteric pocket also had an effect on antagonist action. Removing the structural constraint of the proline residue (conserved in mammalian P2XRs) at position 96 (P96A) decreased antagonist action ∼13-fold for A438079. Mutation of the unique leucine residue at position 97 (L97P; the alanine and cysteine mutants were nonfunctional; Allsopp et al., 2017) produced a 5-fold increase in sensitivity to A438079. Finally, there is a single-amino-acid deletion between residues 99 and 100 unique to the P2X7R. Insertion of an aspartic acid (found in the hP2X1R) at this position (G99in) decreased the sensitivity of A438079 by 14-fold. Taken together these results show that not only the residues that line (are accessible) the base of the allosteric pocket but also those that contribute to the local environment/folding of the base of the pocket are important for antagonist action.

#### A438079 Binding Mode Derived from Ligand Docking and Mutations.

Representative docking poses of the two major clusters from ligand docking of A438079 into the allosteric binding site of hP2X7R were analyzed in detail with respect to the effect of the mutation of individual residues on A438079 antagonism (Fig. 4). Considering that each of the F88A, D92A, T94V, F95A, and F103A mutations renders hP2X7R insensitive to A438079 inhibition, we expected that a realistic docking pose would explain these effects. Although both major clusters located A438079 in the core of the allosteric pocket, poses from cluster 2 were generally in better agreement with the experimental data by placing A438079 in close proximity to T94, F95, and F103. For the representative pose derived from cluster 2, the dichloro-phenyl and tetrazole moieties of A438079 sat deeply in the allosteric pocket and were embedded by the aromatic residues F95 and F103. The pyridine ring of A438079 was oriented more toward the center of the pocket and in proximity to D92 and F88 (Fig. 4B), providing a rationale for how mutation of these residues might affect A438079 antagonism. Taken together this suggested that cluster 2 is the best description of the A438079 binding pose.

## Discussion

This study used chimeras, mutations, and molecular modeling to provide an understanding of subtype-selective antagonist binding at an allosteric hP2X7R binding pocket. It showed that combining advanced ligand-docking techniques and targeted mutations can yield reliable predictions of antagonist binding modes consistent with structural studies. Our study provides information on binding of the antagonist A438079 and places this compound in the middle/base regions of the allosteric pocket. Comparing our data for A438079, A740003, and AZ10606120 (Allsopp et al., 2017) with structural information for A740003 and AZ10606120 (Karasawa and Kawate, 2016) allows detailed characterization of common and unique features of P2X7R allosteric binding modes. We propose that P2X7R-selective antagonism results from a combination of the size of the allosteric pocket determined by residues not in direct interaction with the antagonists and direct interactions of the ligand with the receptor.

A common theme is the similar effects of mutations of residues at the middle and base of the allosteric pocket. Residues displaying the most significant effects common to all three antagonists are F88 and D92. The crystal structures suggest that F88 probably makes hydrophobic/aromatic interactions with aromatic ring systems of AZ10606120 and A740003 (Karasawa and Kawate, 2016), and a similar interaction is seen in the docked pose for A438079. As F88 is well conserved, it does not explain receptor subtype specificity, but mutation data and modeling suggest that this residue is a major contributor to antagonist affinity. As the side chain of D92 is not directly pointing toward the allosteric pocket, or directly interacting with antagonists, we analyzed in more detail its side chain interaction with Y298, an H-bond between the D92 carboxyl and the Y298 hydroxyl group that connects *α*-helix 90–93 with *β*-strand Y291-E301 of the adjacent subunit. Comparing molecular dynamics simulation of wild-type and D92A hP2X7Rs suggests that the disruption of the D92/Y298 interaction leads to destabilization of the *β*-strand Y291-E301 that aligns the allosteric pocket. The destabilization is particularly noticeable at the entrance region as indicated by the fraction of frames in which residues form a *β*-strand. For residues Y299, K300, and E301 the D92A mutation reduces the participation of these residues in the *β*-strand from 98% ± 2%, 85% ± 6%, and 47% ± 29% to 44% ± 29%, 23% ± 13%, and 24% ± 21%, respectively. These data suggest an indirect effect of the D92A mutation on the allosteric pocket rather than direct involvement of the aspartic acid side chain in antagonist binding.

Residues T94, F95, F103, M105, and F108 also contribute to antagonist binding. However, it is important to stress that the pattern between different inhibitors is not fully consistent at these residues and probably reflects subtle differences in binding modes. We propose that these mutations can serve as a “finger print” for variants of allosteric P2X7R inhibition. At the 89–94 chimeras there was a >300-fold decrease in affinity for A438079 and AZ10606120 at the 89–94 chimera, but only a 3-fold decrease for A740003. Considering the effect of the D92A mutation, it is surprising that the 89–94 chimera and T94V mutation have little effect on A740003 action compared with other allosteric inhibitors. Molecular dynamics simulations of P2X7R indicate that T94V mutation affects structure and dynamics of *α*-helix 90–93 (Allsopp et al., 2017) and hence the allosteric pocket. This effect is evident in the 89–94 chimera and T94V mutation for A438079 and AZ10606120, but the A740003 antagonist is distinctively more tolerant to these and other mutational changes than the other inhibitors. This greater tolerance for changes in the case of A740003 indicates that a combination of different sections of the receptor is more important in providing the high affinity binding environment than individual residues. A740003 sits deepest in the pocket, and in the pdP2X7R structure (PDB: 5U1U) the three molecules of A740003 interact with each other in the upper vestibule mediated by their dimethoxy-phenyl moieties. This feature is not found in the docking pose for A438079 and the pdP2X7R X-ray structure with AZ10606120, and it may contribute to the generally comparatively smaller effects of mutations in the case of A740003.

Another difference of interactions is deep in the base of the allosteric pocket. AZ10606120 (PDB: 5U1W) sits less deep in the allosteric pocket compared with A740003 (PDB: 5U1U) and our docked poses for A438079. Here P2X7R has a paralog-specific deletion of one residue. As expected from experimental structures and ligand docking, inserting an aspartic acid between G99 and N100 to render hP2X7R more hP2X1-like has little effect on the binding of AZ10606120 but decreases the affinity of both A740003 and A438079 ∼10-fold.

Although binding to the core of the allosteric site is common to all compounds studied, there are specific differences in binding modes and interactions. For instance, mutations at the entrance to the binding pocket have a greater impact on AZ10606120 sensitivity. For example, K110Y, which would remove bulk, increased AZ10606120 sensitivity but had no effect on A740003 and A438079. This is consistent with the experimentally determined AZ10606120 binding pose (PDB: 5U1W) (Karasawa and Kawate, 2016) that expands toward the entrance region of the allosteric pocket, whereas this is not the case for A740003 (PDB: 5U1U) and our docked poses for A438079 that have limited interactions with the entrance region.

One interesting feature from the chimeras was that changes in the left flipper (chimera 279–285, distant from the allosteric pocket) resulted in a 10- and 30-fold decrease in antagonist action for A740003 and A438079, respectively. However the chimera 279–285 had no effect on the sensitivity to the antagonist AZ10606120 (Allsopp et al., 2017). Interestingly, this chimera increased sensitivity to ATP ∼30-fold (Allsopp et al., 2017) and swapping with the equivalent region from the P2X4R increased potency ∼15-fold (this study). Movement of the left flipper has been proposed to be coupled to channel gating upon ATP binding (Zhao et al., 2014; Karasawa and Kawate, 2016; Wang et al., 2017). Investigating the effect of individual residues in the region 279–285 reveals that the D280A mutation renders the P2X7R 15-times more sensitive to ATP. hP2X7R MD simulations suggest that D280 (a residue unique to P2X7R paralogs) can form a salt bridge to K193 (a conserved residue in close proximity to the ATP binding site). This salt bridge, which is present in about 60% of the MD frames, restricts the movement of the left flipper and anchors the side chain of K193 in a position from which it would probably not contribute to ATP binding. Releasing this restriction would allow K193 to assume its “normal” role in ATP binding and could explain why the D280A mutation increases ATP potency, and hence how D280 contributes to the lower potency of ATP toward P2X7R compared with other P2XRs. We also note that the left flipper is in direct spatial and sequential proximity to *β*-strand Y291-K300 that separates the ATP binding site from the base of the allosteric pocket. A subtle change in the orientation of this strand triggered by the D280A mutation would affect the size and shape of the base region of the allosteric pocket. As A438079 and A740003 sit slightly deeper in the pocket than AZ10606120, such a change in the lower base region of the allosteric pocket should have less effect on binding of AZ10606120.

The crystal structures of the pdP2X7R with allosteric antagonists bound show a more open/looser association of the subunits than available structures for the P2X3R and 4R in the closed state and this gives rise to a larger allosteric binding pocket (Karasawa and Kawate, 2016). Crystal structures are “snapshots” of the receptor and correspond to state(s) that are stable under particular experimental conditions. There is evidence from the P2X1R that cysteine mutants in the upper vestibule are more accessible to MTSEA-biotinylation than predicted from homology models with closed zfP2X4R as a basis (Roberts et al., 2012). This suggests the P2X1R spends some time “at rest” in a more relaxed configuration, with potentially a larger allosteric pocket. However, the amount of time it spends in this state remains to be determined (if it is less than P2X7R, then that would decrease apparent affinity/access), or to what dimensions the pocket increases in this “relaxed” apo state. Our results suggest that for the P2X7R a substantial component underlying the selectivity of antagonists for this receptor results from the availability/open nature of the intersubunit allosteric pocket.

In summary our work shows that computer-based docking can make useful predictions about ligand binding sites at the P2X7R. Given the improvements in templates (with pdP2X7R structure as a basis), this suggests that in silico docking may provide a useful means for identifying novel P2X7R antagonists. The toolbox of P2X7R mutants allows conformation of allosteric binding in the first instance, and detailed characterization of allosteric binding modes to support structure based drug-design. The work also highlighted the contribution of the left flipper to ligand action. Of particular interest was identifying the role of a unique aspartic acid residue that makes a substantial contribution to the reduced ATP potency at the P2X7R.

## Abbreviations

A438079: 3-(5-(2,3-dichlorophenyl)-1H-tetrazol-1-yl)methyl pyridine hydrochloride hydrate
A740003: *N*-[1-[[(cyanoamino)(5-quinolinylamino)methylene]amino]-2,2-dimethylpropyl]-3,4-dimethoxybenzeneacetamide
AZ10606120: *N*-[2-[[2-[(2-hydroxyethyl)amino]ethyl]amino]-5-quinolinyl]-2-tricyclo[3.3.1.13,7]dec-1-ylacetamide dihydrochloride
DMPC: dimyristoylphosphatidylcholine
pd: panda
P2XR: P2X receptor
